# MCC950, a Selective NLRP3 Inhibitor, Attenuates Adverse Cardiac Remodeling Following Heart Failure Through Improving the Cardiometabolic Dysfunction in Obese Mice

**DOI:** 10.3389/fcvm.2022.727474

**Published:** 2022-05-12

**Authors:** Menglong Wang, Mengmeng Zhao, Junping Yu, Yao Xu, Jishou Zhang, Jianfang Liu, Zihui Zheng, Jing Ye, Zhen Wang, Di Ye, Yongqi Feng, Shuwan Xu, Wei Pan, Cheng Wei, Jun Wan

**Affiliations:** ^1^Department of Cardiology, Renmin Hospital of Wuhan University, Wuhan, China; ^2^Cardiovascular Research Institute, Wuhan University, Wuhan, China; ^3^Hubei Key Laboratory of Cardiology, Wuhan, China

**Keywords:** metabolic remodeling, MCC950, inflammasome, pressure overload, cardiac hypertrophy, heart failure

## Abstract

Obesity is often accompanied by hypertension. Although a large number of studies have confirmed that NLRP3 inhibitors can improve cardiac remodeling in mice with a normal diet, it is still unclear whether NLRP3 inhibitors can improve heart failure (HF) induced by pressure overload in obese mice. The purpose of this study was to explore the role of MCC950, a selective NLRP3 inhibitor, on HF in obese mice and its metabolic mechanism. Obese mice induced with a 10-week high-fat diet (HFD) were used in this study. After 4 weeks of HFD, transverse aortic constriction (TAC) surgery was performed to induce a HF model. MCC950 (10 mg/kg, once/day) was injected intraperitoneally from 2 weeks after TAC and continued for 4 weeks. After echocardiography examination, we harvested left ventricle tissues and performed molecular experiments. The results suggest that in obese mice, MCC950 can significantly improve cardiac hypertrophy and fibrosis caused by pressure overload. MCC950 ameliorated cardiac inflammation after TAC surgery and promoted M2 macrophage infiltration in the cardiac tissue. MCC950 not only restored fatty acid uptake and utilization by regulating the expression of CD36 and CPT1β but also reduced glucose uptake and oxidation *via* regulating the expression of GLUT4 and p-PDH. In addition, MCC950 affected the phosphorylation of AKT and AMPK in obese mice with HF. In summary, MCC950 can alleviate HF induced by pressure overload in obese mice *via* improving cardiac metabolism, providing a basis for the clinical application of NLRP3 inhibitors in obese patients with HF.

## Introduction

Heart failure (HF) is a very common progressive disease today, with high morbidity and mortality. Most patients require hospitalization, which puts continuous pressure on the clinical and public health systems. The main risk factors for HF include coronary heart disease, hypertension, diabetes, and obesity (1). Studies have shown that obese patients with a higher body mass index (BMI) have a higher risk of HF (2, 3). A higher BMI is associated with a higher risk of hospitalization and death with HF (4). In addition, visceral fat accumulation is significantly associated with poor cardiac remodeling (5). The metabolic derangements caused by obesity are believed to be closely related to the development of HF. As shown in animal experiments, obesity induced by a high-fat diet (HFD) can aggravate cardiac remodeling (6, 7). And attenuating the metabolic disorder induced by obesity can effectively reduce cardiac remodeling (8). These studies suggest that reducing obesity-induced metabolic disorders may be one of the effective strategies for the treatment of HF. At present, many drugs have been reported to play the role of attenuating cardiac remodeling and HF in mice with a normal diet. However, it is still unclear whether the same cardioprotective effect is available in obese mice.

It has been reported that changes in cardiac metabolism precede the occurrence of HF (9). Damage to cardiac metabolism increases cardiomyocyte death and exacerbates pathological cardiac remodeling. Insufficient ATP production directly changes contractile function and leads to HF. The fatty acid (FA) sources of ATP decrease, while glycolysis and other forms of metabolism source of ATP increase in a failed heart (10). This process is called metabolic reprogramming and is accompanied by changes in genes encoding proteins related to mitochondrial function (11). Moreover, cardiometabolic reprogramming is considered to be the direct cause of pathological hypertrophy (12). Relieving mitochondrial dysfunction and metabolic remodeling have been reported to attenuate the development of pathological hypertrophy and cardiac systolic dysfunction (13, 14). These findings provide new ideas for the treatment of cardiac remodeling and HF.

Nod-like receptor family pyrin domain-containing 3 (NLRP3) inflammasome is a multi-protein complex composed of NLRP3, speck-like protein (ASC), and pro-caspase-1. NLRP3 inflammasome is mainly activated by pathogen-related molecular patterns and danger-related molecular patterns, and further activates caspase-1, thereby promoting the maturation of proinflammatory cytokines IL-1β and IL-18 (15). NLRP3 inflammasome plays an important role in the occurrence and prognosis of HF. A clinical trial involving 54 HF patients reported that compared with the control group, the ASC methylation in the exercise group increased, and the plasma IL-1β and ASC mRNA levels decreased, suggesting that exercise can improve the prognosis of HF through epigenetic regulation of ASC (16). In addition, NLRP3 inflammasome is involved in pathological processes leading to HF, such as cardiac hypertrophy and fibrosis. In the pressure overload model, NLRP3 inflammasome is activated, and inhibiting the expression or activation of NLRP3 can effectively alleviate cardiac remodeling caused by pressure overload (17, 18). In addition, many previous studies have shown that there is a close interaction between NLRP3 and metabolism (19, 20). β-Hydroxybutyrate (BHB), a kind of ketone body, inhibits the activation of NLRP3 inflammasome by preventing K^+^ efflux and reducing ASC oligomerization and speck formation (21). *In vivo*, BHB or ketogenic diet attenuated caspase-1 activation and IL-1β secretion in the mouse models of NLRP3-mediated disease. Increasing the BHB levels attenuates NLRP3 inflammasome formation and antagonizes proinflammatory cytokine-induced mitochondrial dysfunction and fibrosis, thereby improving HF (22). This suggests that NLRP3 may be involved in cardiometabolic reprogramming. However, there is currently a lack of relevant evidence to prove the role of NLRP3 in cardiac metabolic reprogramming in obese mice. Therefore, we used MCC950, a selective inhibitor of NLRP3, to explore the effect of NLRP3 on cardiometabolic reprogramming during HF in obese mice.

## Materials and Methods

### Reagents

MCC950 and wheat germ agglutinin (WGA, 1:200 dilution) was purchased from Sigma-Aldrich (St. Louis, MO, United States). GAPDH (1:2,500 dilution), α-smooth muscle actin (α-SMA, 1:500 dilution), CD68 (1:200 dilution), CD206 (1:200 dilution), CD36 (1:500 dilution), glucose transporter 4 (GLUT4, 1:500 dilution) were purchased from Abcam (Cambridge, MA, United States). Collagen 1 (COL1, 1:1,000 dilution), COL3 (1:1,000 dilution), carnitine palmitoyl transferase 1β (CPT1β, 1:1,000 dilution), total acetyl-CoA carboxylase (t-ACC, 1:1,000 dilution), phosphorylated-ACC (Ser79) (p-ACC, 1:1,000 dilution), malonyl-CoA decarboxylase (MCD, 1:1,000 dilution), GLUT1 (1:1,000 dilution), total pyruvate dehydrogenase (t-PDH, 1:1,000 dilution), phosphorylated-PDH (Ser232) (p-PDH, 1:1,000 dilution), pyruvate dehydrogenase kinase 4 (PDK4, 1:1,000 dilution) were purchased from Proteintech (Wuhan, China). Caspase-1 (1:500 dilution) and interleukin 1β (IL1β; 1:500 dilution) were purchased from Santa Cruz (United States). Total protein kinase B (t-AKT, 1:1,000 dilution), phosphorylated-AKT (Ser473) (p-AKT, 1:1,000 dilution), total AMP-activated protein kinase α (t-AMPKα, 1:1,000 dilution), and phosphorylated-AMPKα (Thr172) (p-AMPKα,1:1,000 dilution) were purchased from Cell Signaling Technology (United States). Secondary antibodies were obtained from LI-COR Biosciences (Lincoln, NE, United States). All other chemicals were of analytical grade. The catalog numbers are provided in Supplementary Table 1.

### Animal Models

C57BL/6J mice (6 weeks, males, Gempharmatech Co., Ltd.) in this study were raised in a specific pathogen-free mouse room in the Renmin Hospital of Wuhan University. The room provided the mice with stable temperature (20–22°C), humidity (50 ± 5%), plenty of water, and food for free drinking and eating. We performed mouse experiments and took care following the Guidelines for the Care and Use of Laboratory Animals published by the National Institutes of Health. The Animal Care and Use Committee of Renmin Hospital of Wuhan University (Wuhan, China) has reviewed and approved this research.

At 8 weeks of age, the mice were initiated with a HFD, containing 60% fat by kcal content (product D12492, Research Diets, New Brunswick, NJ, United States) to produce obese models (23). We divided these mice into four groups based on the treatment given: sham + vehicle, sham + MCC950, TAC + vehicle, TAC + MCC950 (*n* = 10, per group). The mice had *ad libitum* access to food and water during the whole experiments. And 4 weeks after HFD, the transverse aortic constriction (TAC) or sham surgery was performed. Briefly, the mice were anesthetized with 2% isoflurane inhalation. The mice were then placed in the supine position and orally intubated with a 20-gauge tube and ventilated (Harvard Apparatus Rodent Ventilator, Model 845) at 120 breaths per minute (0.1 ml tidal volume). The thoracic cavity was exposed by incising the proximal portion of the sternum. After isolating the aortic arch between the innominate and left common carotid arteries, the transverse aortic arch was ligated with a covered 26-gauge needle. Then the needle was removed, leaving a discrete area of stenosis. The sham-operated mice underwent the same surgical procedure, including isolation of the aorta, but no sutures were placed. Normal saline was intraperitoneally injected as a vehicle. MCC950 or vehicle (10 mg/kg of body weight, once/day) was intraperitoneally injected into the mice 2 weeks after the TAC or sham surgery (24, 25). HFD was maintained after surgery for 6 weeks for heart mass, cardiac function, and other analyses. And the left ventricle (LV) tissue was harvested for further experiments. The design of this study is shown in Supplementary Figure 1.

### Echocardiography

Mylab 30CV ultrasound (Biosound Esaote) equipped with a 10-MHz linear array ultrasound transducer was used to perform echocardiography in the anesthetized (1.5–2% isoflurane) mice. The LV dimensions were assessed at the parasternal short axis at the level of the papillary muscles. The LV end-systolic diameter (LVEDs), LV end-diastolic diameter (LVEDd), interventricular septal thickness at end-diastole (IVSd), interventricular septal thickness at end-systole (IVSs), LV posterior wall thickness at end-diastole (LVPWd), LV posterior wall thickness at end-systole (LVPWs), LV ejection fraction (EF), and fractional shortening (FS) were measured.

### Histological Analysis

There were five samples per group for histological analysis. We took four images of each sample for quantification. Hematoxylin-eosin (H&E) and WGA staining were performed to examine the size of cardiomyocytes. Masson staining was used to observe collagen deposition in the perivascular space and interstitium of the four groups of mice. We determined the cross-sectional area of cardiomyocytes with WGA staining and the fibrotic areas with Masson staining by using a digital image analysis system (Image-Pro Plus 6.0, Media Cybernetics, Bethesda, MD, United States). In WGA staining, we selected seven cardiomyocytes in each image to measure the cardiomyocyte size. In Masson staining, we examined the ratio of collagen fibers to the cross-sectional area of the ventricle. Immunohistochemistry and immunofluorescence staining were performed to examine the macrophage infiltration as per the protocols mentioned in a previous study (26). We examined the percentage of positive cells in immunohistochemical staining for aSMA, CD68, and immunofluorescence staining for CD206 using Image J 2.1.0.

### Quantitative Polymerase Chain Reaction

The RNA was extracted from the LV tissue using TRIzol (Invitrogen Life Technologies, Carlsbad, CA, United States), and cDNA was synthesized using 2 μg RNA of each treatment group with oligo (dT) primers and a Transcriptor First Strand cDNA Synthesis Kit (Roche, Basel, Switzerland). Quantitative analysis was performed using a Light Cycler 480 and SYBR Green Master Mix (Roche). Details of the primer sequences are shown in Supplementary Table 2.

### Measurement of Blood Parameters

After a 12-h fast, blood was collected from the retro-orbital plexus to measure the serum levels of blood glucose, triglyceride (TG), non-esterified fatty acids (NEFAs), and BHB following the instructions. All the detection kits were purchased from Nanjing Jiancheng Bioengineering Institute, China.

### Measurement of Triglyceride, Glycogen, and Pyruvate Dehydrogenase Activity in Hearts

The ventricles were immediately snap-frozen in liquid nitrogen and pulverized with a mortar and pestle in liquid nitrogen. TG and glycogen concentrations in the heart, as well as PDH activity, were then determined according to the manufacturer’s instructions. All the detection kits were purchased from Nanjing Jiancheng Bioengineering Institute, China.

### Western Blotting

We extracted proteins from the cardiac tissue using radioimmunoprecipitation assay (RIPA) buffer and measured protein concentration using the BCA protein assay kit. The proteins (50 μg) were separated by sodium dodecyl sulfate-polyacrylamide gel electrophoresis (SDS-PAGE) and subsequently transferred to Immobilon-P membranes (Millipore, Beijing, China) by a gel transfer apparatus (Invitrogen). The membranes were incubate with different primary antibodies over night at 4°C. After washing, the membranes were incubated with secondary antibodies for 1h at room temperature. The blots were scanned by a two-color infrared imaging system (Odyssey; LI-COR) to quantify the protein expression. The protein expression levels were normalized to the corresponding GAPDH levels.

### Statistical Analysis

The results were presented as mean ± standard deviation (SD). Statistical differences between two groups were compared using Student’s *t*-tests, and the differences between multiple groups were compared by two-way analysis of variance (ANOVA), followed by Tukey’s multiple comparisons test. All the data were analyzed using SPSS 26.0 software, and a *p*-value < 0.05 was considered statistically significant and was the threshold used to reject the null hypothesis.

## Results

### MCC950 Alleviated Cardiac Dysfunction Induced by Transverse Aortic Constriction Surgery in Obese Mice

**FIGURE 1:**
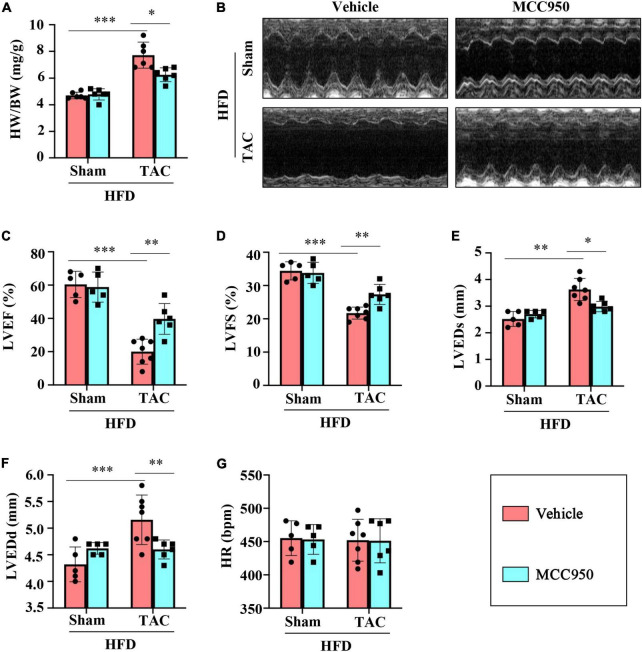
MCC950 reversed the cardiac dysfunction post-TAC. **(A)** The ratio of heart weight (HW)/body weight (BW) in different groups, *n* = 6. **(B)** Representative images of echocardiography. **(C–G)** Echocardiography measurements of LVEDd, LVEDs, LVEF, LVFS, and HR, respectively, *n* = 6. LVEF, left ventricle ejection fraction; LVFS, left ventricle fractional shortening; LVEDs, LV end-systolic diameter; LVEDd, LV end-diastolic diameter; HR, heart rate. *p < 0.05, ^**^p < 0.01, ^***^p < 0.001.

The ratio of heart weight (HW)/body weight (BW) increased after the TAC surgery, which revealed the compensatory cardiac weight gain (Figure 1A). MCC950 reversed these increases significantly. Six weeks after the TAC surgery, the cardiac function of the obese mice was significantly impaired, as shown by the decreased LVEF and LVFS and increased LVEDd and LVEDs (Figures 1B–F and Supplementary Table 3). MCC950 treatment increased LVEF and FS and reduced LVEDd and LVEDs after TAC. There was no difference in the heart rate (HR) between the four groups (Figure 1G). Taken together, MCC950 alleviated cardiac dysfunction induced by TAC surgery.

### MCC950 Attenuated the Cardiac Hypertrophy Induced by Transverse Aortic Constriction Surgery in Obese Mice

**FIGURE 2:**
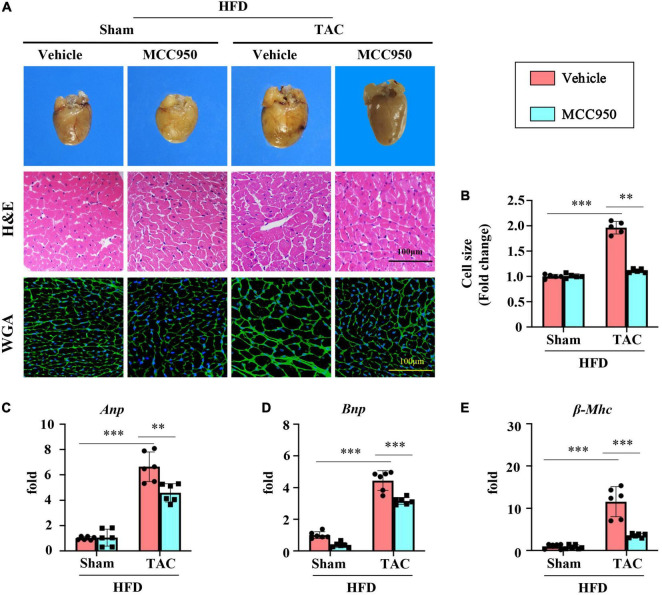
MCC950 alleviated pressure overload-induced cardiac hypertrophy. **(A)** Representative photographs presenting morphology of the hearts, representative images of hematoxylin-eosin (H&E), and WGA staining for the determination of cardiomyocyte cell size, *n* = 5. **(B)** Cardiac hypertrophy was quantified by measuring the cross-sectional cell-surface area of cardiomyocyte from WGA staining, *n* = 5. **(C–E)** Q-PCR to measure mRNA expression of ANP, BNP, and β-MHC, respectively; *n* = 6. H&E, hematoxylin-eosin; WGA, wheat germ agglutinin; ANP, atrial natriuretic peptide; BNP, brain natriuretic peptide; β-MHC, β-myosin heavy chain. *p < 0.05, ^**^p < 0.01, ^***^p < 0.001.

Cardiac hypertrophy was induced by TAC surgery as shown by the gross morphology and coronal sections of the heart (Figure 2A). The size of cardiomyocytes increased significantly after TAC surgery as shown by the H&E and WGA staining (Figures 2A,B). The MCC950 treatment reversed the increased size of heart and cardiomyocytes induced by pressure overload (Figures 2A,B). In addition, MCC950 reduced the TAC-induced increased mRNA expression level of the genes related to cardiac hypertrophy, including atrial natriuretic peptide (ANP), brain natriuretic peptide (BNP), and β-myosin heavy chain (β-MHC) (Figures 2C–E). These results revealed that MCC950 attenuated the pressure-overload-induced cardiac hypertrophy in obese mice.

### MCC950 Ameliorated the Cardiac Fibrosis Induced by Pressure Overload in Obese Mice

**FIGURE 3:**
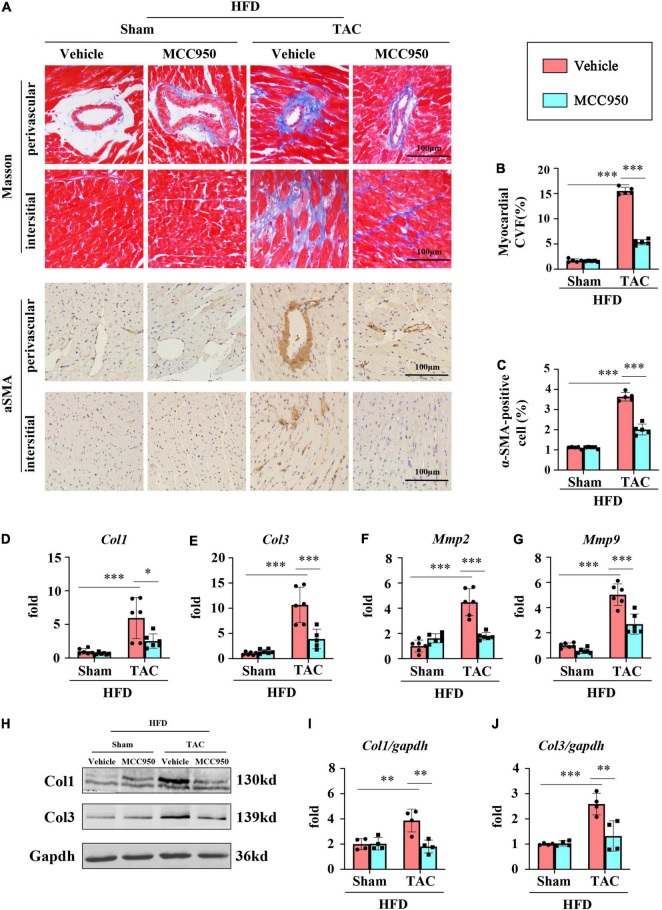
MCC950 ameliorates pressure overload-induced cardiac fibrosis. **(A)** Representative images of Masson’s trichrome and αSMA immunohistochemistry staining, *n* = 5. **(B)** Statistical results for the fibrotic areas in the indicated groups, *n* = 5. **(C)** Statistical results for the αSMA positive cells in the indicated groups, *n* = 5. **(D)** Relative mRNA expression of COL1, **(E)** COL3, **(F)** MMP-2, **(G)** MMP-9, *n* = 6. **(H–J)** Representative Western blot and quantitative analysis showing the expression levels of COL1 and COL3. αSMA, α-smooth muscle actin; COL, collagen; MMP, matrix metalloproteinase. *p < 0.05, ^**^p < 0.01, ^***^p < 0.001.

To validate the cardiac fibrosis induced by pressure overload, masson staining was conducted. Cardiac fibrosis was found around the peripheral blood vessels and in the myocardial interstitium, which was attenuated by the MCC950 treatment (Figures 3A,B). MCC950 also reduced the expression of α-SMA after TAC surgery (Figures 3A,C). Besides, the mRNA expression of COL1 and COL3 increased significantly after TAC surgery. MCC950 reversed this increase (Figures 3D,E). In addition, MCC950 attenuated the increased expression level of matrix metalloproteinase 2 (MMP-2) and MMP-9 induced by pressure overload (Figures 3F,G). MCC950 also reduced the protein levels of COL1 and COL3 after TAC surgery (Figures 3H–J). Together, these data indicated that MCC950 ameliorated the cardiac fibrosis induced by pressure overload in obese mice.

### MCC950 Attenuated the Cardiac Inflammation Induced by Transverse Aortic Constriction Surgery in Obese Mice

**FIGURE 4:**
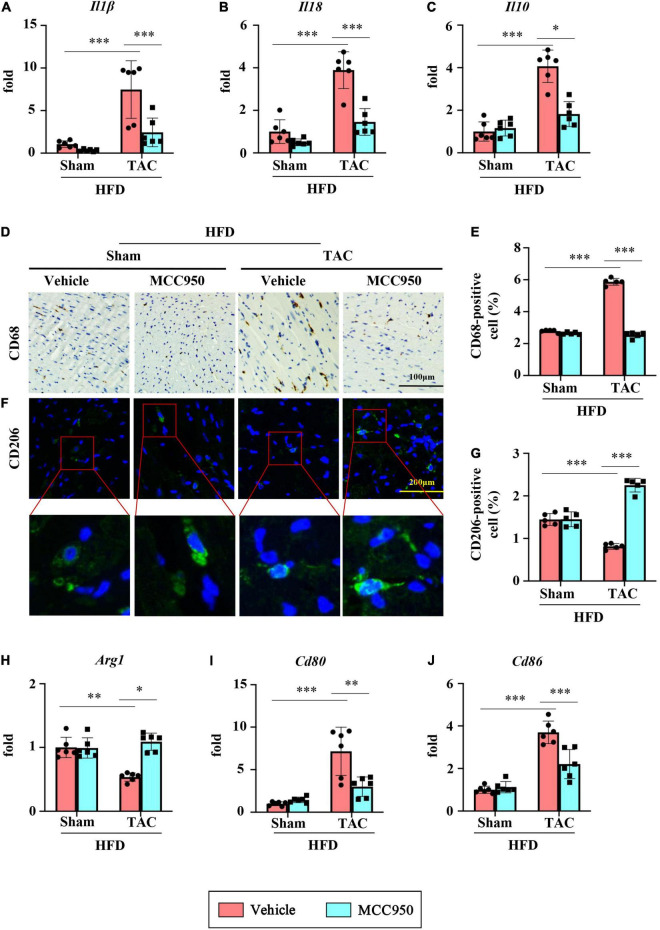
MCC950 attenuates pressure overload-induced cardiac inflammation. Relative mRNA expression of **(A)** IL1β, **(B)** IL18, and **(C)** IL10, *n* = 6. **(D,E)** Immunohistochemical analysis of CD68, *n* = 3. **(F,G)** immunofluorescence analysis of CD206, *n* = 3. **(H–J)** Relative mRNA expression of Arg1, CD80, and CD86, *n* = 6. IL, interleukin; Arg1, arginase 1. *p < 0.05, ^**^p < 0.01, ^***^p < 0.001.

The mRNA expression level of proinflammatory cytokines including IL1β and IL18 increased significantly after TAC surgery (Figures 4A,B). MCC950 reversed these increases. Interestingly, MCC950 also reduced the increased expression of anti-inflammatory cytokine IL10 induced by pressure overload (Figure 4C). In addition, MCC950 reduced the infiltration of macrophages induced by TAC surgery as shown by CD86 immunohistochemical staining (Figures 4D,E). We next examined the polarization of macrophages in the cardiac tissues. The immunofluorescence results showed that MCC950 treatment can significantly increase the expression of CD206, a marker of M2 macrophage (Figures 4F,G). And we also tested the mRNA expression levels of other markers of macrophage polarization. After TAC surgery, the expression of arginase 1 (ARG1, a marker of M2 macrophage) was significantly reduced, and the expressions of CD80 and CD86 (M1 macrophage markers) were significantly increased (Figures 4H–J). MCC950 treatment increased the expression of ARG1 and inhibited the expressions of CD80 and CD86. These results indicate that MCC950 treatment can induce M2 polarization of macrophages. Taken together, MCC950 attenuated the cardiac inflammation induced by pressure overload in obese mice.

### MCC950 Reduced the Metabolic Reliance on Glucose in Failed Hearts

**FIGURE 5:**
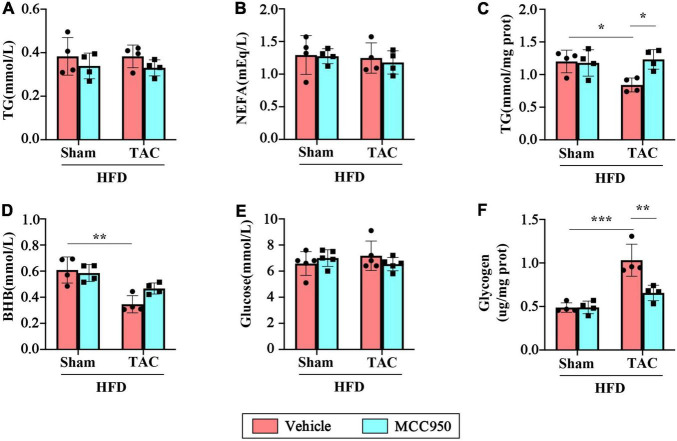
MCC950 reduced the metabolic reliance on glucose in TAC hearts. Serum levels of TG **(A)** and NEFA **(B)**, *n* = 4. **(C)** Level of TG in the cardiac, *n* = 4. Serum levels of BHB **(D)** and glucose **(E)**, *n* = 4. **(F)** glycogen levels in the cardiac, *n* = 4. TG, triglyceride; NEFA, non-esterified fatty acids; BHB, β-hydroxybutyrate. *p < 0.05, ^**^ *p* < 0.01, ^***^ *p* < 0.001.

We next examined the levels of metabolism related biochemical parameters in the serum and heart after TAC surgery. MCC950 did not affect TG and NEFA in the serum of mice after TAC surgery (Figures 5A,B). After TAC surgery, the accumulation of TG, a storage form of FA, in the heart is significantly reduced, suggesting that the uptake of FA by myocardial tissue is reduced (Figure 5C). However, the MCC950 treatment can significantly increase the level of accumulated TG in the heart. The circulating levels of BHB decreased significantly after TAC surgery (Figure 5D). However, the MCC950 treatment did not affect the circulating levels of BHB. We also tested the circulating level of glucose and found that there was no significant change in the serum glucose levels either in TAC surgery or the MCC950 treatment group (Figure 5E). Glycogen, a storage form of glucose, increased significantly in the heart after TAC surgery, indicating that the cardiac tissue heavily relies on glucose after TAC. MCC950 can significantly reduce the accumulation of glycogen in the heart (Figure 5F). These results indicate that MCC950 treatment can reduce the dependence of cardiac tissue on glucose after HF, and at the same time restore the use of FA.

### MCC950 Increased the Gene Expression for Fatty Acid Use in Obese Mice After Transverse Aortic Constriction Surgery

**FIGURE 6:**
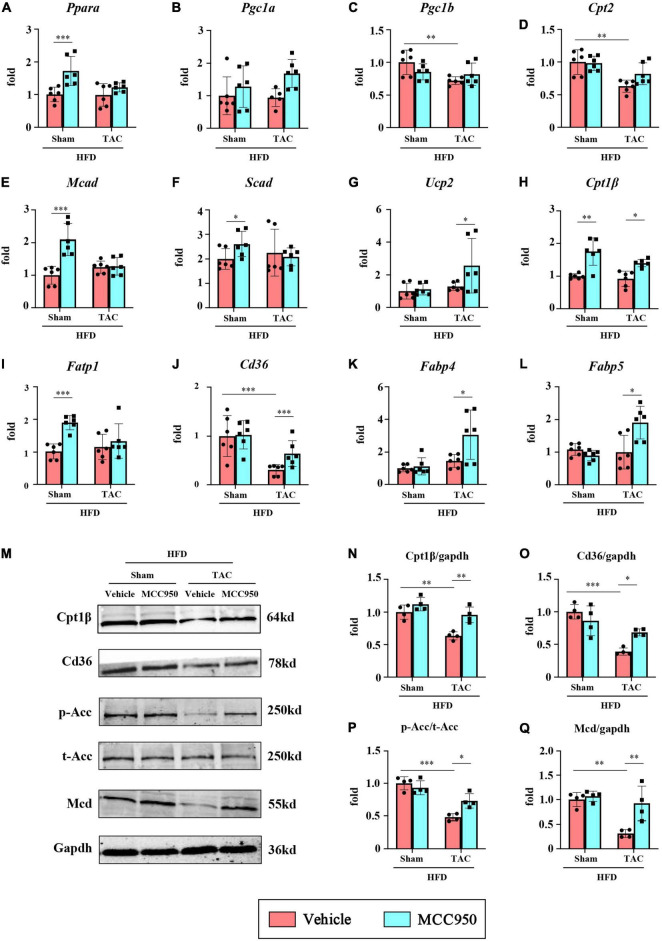
MCC950 increased the gene expression for FA use in obese mice after TAC surgery. Relative mRNA expression of **(A)** PPARα, **(B)** PGC1α, **(C)** PGC1β, **(D)** CPT2, **(E)** MCAD, **(F)** SCAD, **(G)** UCP2, **(H)** CPT1β, **(I)** FATP1, **(J)** CD36, **(K)** FABP4, **(L)** FABP5, *n* = 6. **(M–Q)** Representative blots and quantitative analysis showing the expression levels of CPT1β, CD36, p-ACC, t-ACC, and MCD, *n* = 4. PPAR, peroxisome proliferator-activated receptor; PGC1, PPARγ-coactivator-1; CPT, carnitine palmitoyl transferase; MCAD, medium-chain acyl-CoA dehydrogenase; SCAD, short-chain acyl-CoA dehydrogenase; UCP2, uncoupling protein 2; FATP, FA transport protein; FABP, FA binding protein. ACC, acetyl-CoA carboxylase; MCD, malonyl-CoA decarboxylase. *p < 0.05, ^**^p < 0.01, ^***^p < 0.001.

We first examined the mRNA expression of the genes relevant to FA uptake and oxidation, including peroxisome proliferator-activated receptor α (PPARα) and its co-activators PPARγ-coactivator-1(PGC1) α and β. MCC950 did not change the expression of these three genes in the mice after TAC surgery (Figures 6A–C). Consistent with these findings, MCC950 did not change the expression of the genes regulated by the PPARα/PGC1 complex, including carnitine palmitoyltransferase (CPT) 2, medium-chain acyl-CoA dehydrogenase (MCAD), short-chain acyl-CoA dehydrogenase (SCAD), and uncoupling protein 2 (UCP2) (Figures 6D–G). However, the MCC950 treatment significantly increased the mRNA expression of CPT1β, which plays an important role in the oxidation of FA, after TAC surgery (Figure 6H). We also tested the expression of genes involved in FA uptake, including fatty acid transport protein 1(FATP1) and thrombospondin receptor (CD36) (Figures 6I,J). Compared with the sham group, the mRNA expression of CD36 in the heart of the TAC group was significantly reduced. The MCC950 treatment can alleviate this reduction. This suggests that MCC950 can promote the intake of FA in the heart. In addition, we also tested the expression level of FA binding proteins (FABP) involved in the intracellular transport of FA (Figures 6K,L). MCC950 increased the expression of FABP4 and FABP5 in the hearts of mice in the TAC group, suggesting that it promoted the transport and oxidation of intracellular FA.

We further tested the protein expression levels of CPT1β and CD36 (Figures 6M–O). Compared with the sham group, the expression levels of CPT1β and CD36 in the heart of the TAC group were significantly reduced, and MCC950 significantly reversed this reduction. The key site regulating FA oxidation is the inhibition of CPT1β by malonyl-COA. The synthesis of malonyl-COA is regulated by ACC and the degradation is controlled by MCD. We tested the protein levels of ACC and MCD (Figures 6P,Q). After TAC surgery, p-ACC in the mouse heart was significantly reduced, suggesting an increase in ACC activity. Compared with mice in the sham group, the expression of MCD in the heart of the TAC group was reduced. The MCC950 treatment can significantly increase the phosphorylation level of ACC and increase the protein level of MCD. These results indicate that MCC950 can restore the uptake and oxidation of FA in the cardiac tissue after HF in obese mice.

### MCC950 Regulated the Gene Expression Relevant to Glucose Metabolism in Obese Mice After Transverse Aortic Constriction Surgery

**FIGURE 7:**
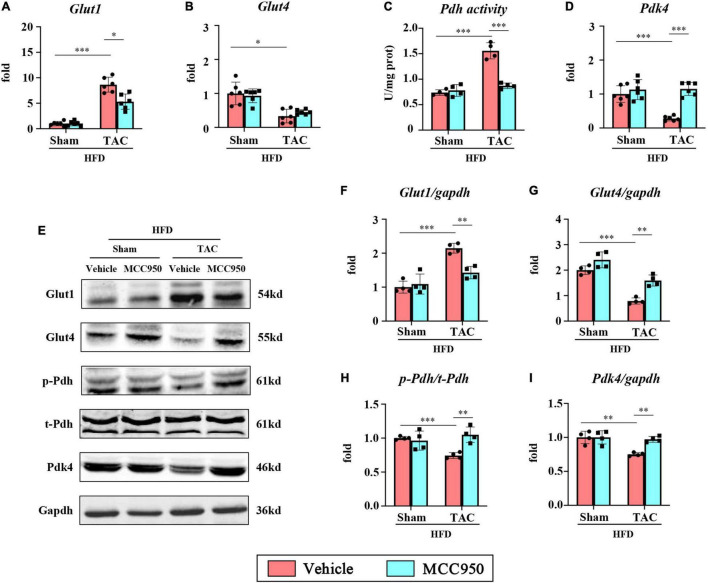
MCC950 regulated the gene expression relevant to glucose metabolism in obese mice after TAC surgery. Relative mRNA expression of **(A)** GLUT1 and **(B)** GLUT4, *n* = 6. **(C)** PDH activity in the cardiac tissue, *n* = 4. **(D)** Relative mRNA expression of PDK4, *n* = 6. **(E–I)** Representative blots and quantitative analysis showing the expression levels of GLUT1, GLUT4, p-PDH, t-PDH, and PDK4, *n* = 4. GLUT, glucose transporter; PDH, pyruvate dehydrogenase; PDK, pyruvate dehydrogenase kinase. *p < 0.05, ^**^p < 0.01, ^***^p < 0.001.

We then examined the mRNA expression of the gene relevant to glucose metabolism. In the heart, the most abundant GLUTs are GLUT1 and GLUT4, which are of great significance for the uptake and transport of glucose. The mRNA expression of GLUT1 increased, while the expression of GLUT4 decreased after TAC surgery (Figures 7A,B). Interestingly, MCC950 reversed the increase of GLUT1 induced by pressure overload. Consistently, the total protein expression levels of GLUT1 increased, while GLUT4 decreased after TAC surgery (Figures 7E–G). MCC950 reduced the level of GLUT1 and increased the level of GLUT4. Glycolysis increases the supply of pyruvate for mitochondrial glucose oxidation through the PDH complex, the rate-limiting enzyme for glucose oxidation. The activity of PDH in the cardiac tissue increased significantly after TAC surgery and recovered after the MCC950 treatment (Figure 7C). The activity of PDH is mainly regulated by its phosphorylation state and is active when it is dephosphorylated. Consistently, the ratio of p-PDH to t-PDH is significantly reduced after TAC surgery, and the MCC950 treatment can promote PDH phosphorylation (Figure 7H). The PDH complex can be phosphorylated and inhibited by pyruvate dehydrogenase kinase (PDK). Our results suggest that the mRNA expression level and protein expression level of PDK4 are significantly reduced after TAC surgery, and MCC950 can effectively promote the expression of PDK4 (Figures 7D,I). These results suggest that MCC950 can regulate the expression of genes related to glucose metabolism after HF in obese mice.

### MCC950 Attenuated Cardiometabolic Disorder *via* the Protein Kinase B/AMP-Activated Protein Kinase α Pathway

**FIGURE 8:**
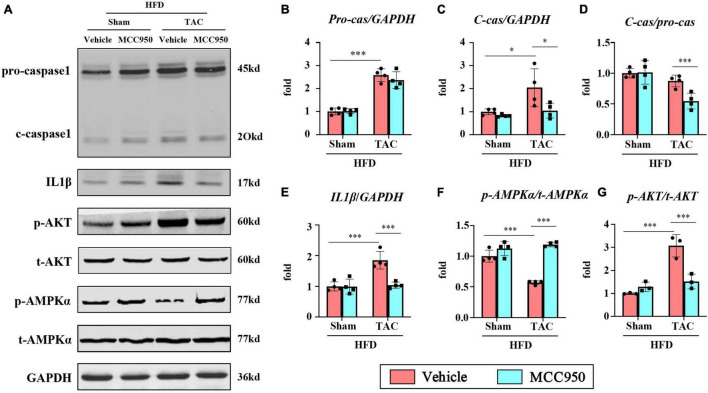
MCC950 attenuated cardiometabolic disorder *via* AKT/AMPK pathway. **(A)** Representative Western blot and quantitative analysis showing the expression levels of **(B)** pro-caspase-1, **(C)** c-caspase-1, **(D)** the ratio of c-caspase-1/pro-caspase-1, **(E)** IL-1β, **(F)** p-AMPKα, and **(G)** p-AKT in different groups, *n* = 4. IL, interleukin; AKT, phospho-protein kinase B; AMPKα, AMP-activated protein kinase α. *p < 0.05, **p < 0.01, ***p < 0.001.

**FIGURE 9:**
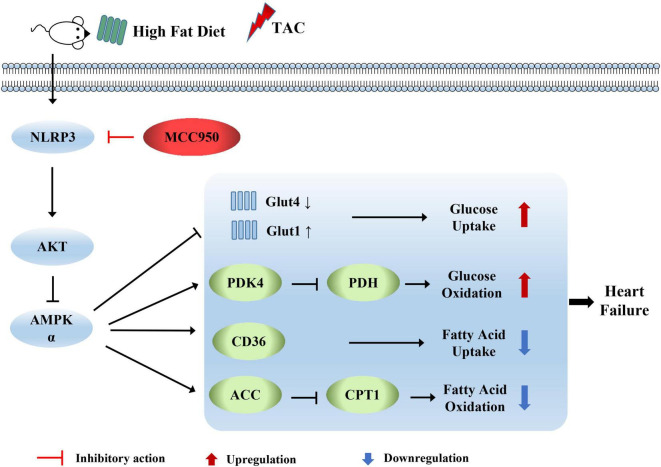
Diagram depicting the mechanism by which MCC950 regulates cardiometabolism in obese mice with heart failure.

We first examined the inhibitory effect on the activation of NLRP3 of MCC950. The expression level of c-caspase-1 and IL-1β increased after pressure overload, suggesting that the NLRP3 inflammasome was activated. The selective inhibitor of NLRP3, MCC950, significantly reversed these increases (Figures 8A–E). AMPK acts as an energy sensor to regulate multiple physiological processes in the cardiovascular system. Previous studies have shown that AMPK can improve HF by restoring energy supply and mitochondrial function (27). We detected the level of t-AMPKα and p-AMPKα. The level of p-AMPKα decreased after TAC surgery and increased with MCC950 treatment (Figure 8F). In addition, we also examined the phosphorylation level of AKT, an upstream regulator of AMPKα. The ratio of p-AKT to t-AKT increased after TAC surgery while it decreased with the MCC950 treatment (Figure 8G). Taken together, these results suggested that MCC950 attenuated cardiac metabolic disorder *via* AKT/AMPKα pathway (Figure 9).

## Discussion

In the present study, MCC950 significantly ameliorated the cardiac dysfunction, hypertrophy, and fibrosis induced by pressure overload in obese mice. The increased inflammation after TAC surgery was attenuated by the MCC950 treatment. Besides, MCC950 reversed the cardiac metabolic disorder *via* AKT/AMPK pathway as shown by the regulation of glucose and FA uptake and oxidation. Taken together, these results suggested the treatment effect of MCC950 in obese mice with HF.

### MCC950 Protected Left Ventricle Function and Structure in Obese Mice With Pressure Overload

Many reports indicated that NLRP3 inflammasome may be a new promising effective therapeutic target to slow down the disease progression of HF (28). Our previous study found that MCC950 can reverse HF induced by pressure overload in mice (29). This study further indicated that MCC950 can also effectively improve LV function in obese mice, as shown by improved LVEF and FS. The improvement of cardiac function may be related to the alleviated cardiac remodeling by the MCC950 treatment. MCC950 can significantly inhibit heart enlargement and cardiac hypertrophy. In addition, the MCC950 treatment ameliorated cardiac fibrosis induced by pressure overload, as shown by the alleviated expression of COL1, COL3, and a-SMA. In short, MCC950 protected LV function and structure in obese mice with pressure overload.

### MCC950 Ameliorated Cardiac Inflammation After Transverse Aortic Constriction Surgery in Obese Mice

Inflammatory responses play an important role in the development of HF. IL1β is one of the most important proinflammatory cytokines involved in HF. The link between IL1β and HF is supported by the elevated level of IL1β in patients with worsening HF symptoms and outcomes (30). Direct reduction of IL1β levels or blocking of the downstream signal of IL1β contribute to helpful cardiac remodeling and improvements of HF phenotype induced by ischemia/infarction, pressure overload, or anthracycline toxicity (31). As the maturation of IL1β is regulated *via* the activation of the NLRP3 inflammasome, NLRP3 is considered a potential therapeutics target for HF. The cardiac beneficial effects of some molecules are reported to be relevant to the inhibition of the NLRP3 inflammasome (32, 33). Treatments inhibiting the activation of NLRP3 contributed to improved cardiac function and remodeling in the mice with myocardial infarction and cardiac hypertrophy (18, 34). Corresponding with these reports, MCC950 significantly inhibited the activation of NLRP3, preserved cardiac function, and alleviated the inflammation post pressure overload in obese mice. The expression levels of IL1β and other cytokines were significantly reduced, which may contribute to the improvement of HF phenotype and cardiac remodeling. In addition, the MCC950 treatment induced M2 polarization as shown by the increased expression of CD206 and ARG1. The NLRP3 inflammasome was reported to induce M1 polarization in the previous studies (19). In our study, we did not discuss the mechanism by which MCC950 induced M2 polarization. Studies have shown that macrophage polarization is closely related to cell metabolism (35). Therefore, we speculate that in addition to inhibiting the IL1β expression, MCC950 may also induce M2 polarization by affecting the cell metabolism, which needs further exploration.

### MCC950 Treatment Alleviated the Glucose Dependence of Cardiometabolism in Obese Mice With Heart Failure

Fatty acid is the primary fuel for cardiomyocytes to produce energy in normal adults. But in the development of pathological hypertrophy and HF, cardiomyocytes reduce FA-derived energy and increase glycolysis, anaplerosis, and other forms of metabolism, such as the use of lactate, branched-chain amino acids, and ketone bodies, remodeling ATP production mechanism. This shift in the energy production machinery is known as metabolic reprogramming and is accompanied by the downregulation of genes involved in mitochondrial energy transduction and respiratory pathways (12). In our study, we did not observe the changes in TG, glucose, and NEFA in the serum of obese mice after HF. Only the serum levels of BHB were significantly decreased after HF, suggesting that cardiac utilization of BHB was increased after HF. MCC950 treatment had no apparent effect on the levels of these markers in the serum of obese mice, which may be related to the HFD diet during the experiment. However, we observed decreased TG accumulation and increased glycogen levels in the LV of obese mice after HF, suggesting that mouse cardiometabolism relies more on glucose after HF. Furthermore, the MCC950 treatment increased TG accumulation and decreased glycogen levels in the heart. In a word, MCC950 treatment alleviated the glucose dependence of cardiometabolism and restored FA uptake and utilization.

### MCC950 May Promote Cardiac Fatty Acid Metabolism in Obese Mice With Pressure Overload

In our study, MCC950 did not significantly alter the expression of some genes related to FA metabolism, such as PPARα, PGC1α, and β, CPT2, MCAD, SCAD, and UCP2, which may be related to the HFD in the experiment. HFD has been reported to affect the progression of HF and may also affect the expression of metabolism-related genes (36). However, we observed a marked decrease in the expression of CD36 and CPT1β after HF. CD36 plays an important role in FA uptake in the cardiac. The CD36 knockout mice showed reduced rates of FA transport and oxidation, and significantly increased glucose use in the heart, ultimately exacerbating stress overload-induced HF (37). CPT1β is the rate-limiting step in mitochondrial β-oxidation by controlling mitochondrial uptake of long-chain acyl-CoA. It has been reported that the CPT1β deficiency exacerbates pressure overload-induced cardiac hypertrophy (38). The current findings suggest that MCC950 promoted the expression of CD36 and CPT1β after TAC surgery, implying that MCC950 can promote FA transport and oxidation in the hearts of obese mice with HF. Furthermore, CPT1β is inhibited by malonyl-CoA, the carboxylation product of acetyl-CoA, which is produced by the action of ACC and decarboxylated by MCD (39). In this study, MCC950 increased the phosphorylation level of ACC and increased the expression of MCD, thereby reducing the production of malonyl-CoA and promoting its degradation, ultimately increasing the level of CPT1β.

Modulation of cardiometabolic substrates is one of the strategies for the treatment of HF. However, FA is often discarded due to its high oxygen consumption and potential lipotoxicity (40). Considering that FA produces approximately three times as much ATP per molecule as glucose, it is impossible to rely on glucose alone to provide energy for cardiometabolism. Enhancing FA metabolic preference may be another strategy to restore cardiac energy and function. In our study, MCC950 not only increased the expression of CD36 but also promoted the expression of CPT1β. This means that MCC950 promotes the uptake and oxidation of FA in the heart, which may alleviate the lipotoxicity caused by the accumulation of FA. Taken together, the effect of MCC950 on CD36 and CPT-1β suggests that it may have a benign regulatory effect on cardiac FA metabolism, although it requires further exploration.

### MCC950 Reduced Cardiac Glucose Uptake and Utilization in Obese Mice With Heart Failure

The first step in carbohydrate metabolism involves the uptake of glucose by cardiomyocytes through the action of GLUTs. Glucose transport by these proteins is one of the rate-limiting steps in substrate utilization in the myocardium (41). GLUT4 is insulin-sensitive and is the major isoform in adult myocardium. GLUT1 is insulin-independent and mainly expressed in the fetal heart (39). The GLUT4/GLUT1 ratio has been reported to be reduced in patients with LV hypertrophy (42). Similar observations were made in animal models of pathological cardiac hypertrophy. The pressure overload increased GLUT1 expression but decreased GLUT4 expression, and these changes were associated with increased distribution of both transporters to the plasma membrane (43). Changes from GLUT4 to GLUT1 have been found in failed myocardium, but the mechanism remains unknown (44). Insulin resistance may be one of the reasons why GLUT4 expression is suppressed during HF (45). Therefore, GLUT1 expression may be increased to compensate. In this study, MCC950 significantly downregulated the expression of GLUT1 and restored the content of GLUT4, which may be related to the improvement of insulin resistance after HF by MCC950. The activation of NLRP3 is closely related to insulin resistance in many metabolic diseases (19). In a word, MCC950 promotes glucose uptake in failed hearts in a GLUT4-dependent manner.

Glucose is metabolized by oxidative metabolism under aerobic conditions, or by anaerobic glycolysis in the presence of hypoxia. The pyruvate derived from glucose is converted by PDH to acetyl-CoA for aerobic respiration and is inhibited when phosphorylated by PDH kinase (46). In the mice with HF, the source of cardiac energy changed from FA-dominated to glucose-oxidized-dominant. Consistent with the previous studies, the activity of PDH was significantly increased after TAC surgery. The p-PDH expression and p-PDH/t-PDH ratio were significantly decreased after TAC surgery. The PDK4 expression was significantly reduced in failed hearts. The MCC950 treatment reversed PDH activity and increased PDK4 expression, promoting PDH phosphorylation. These results suggest that the MCC950 treatment reduces glucose oxidation, which may be associated with the restoration of FA metabolism.

### MCC950 Attenuated Cardiometabolic Disorder *via* Protein Kinase B/AMP-Activated Protein Kinase α Pathway in Obese Mice

The changes in AKT activity are an alteration in pathological cardiac hypertrophy. AKT activation not only stimulates protein synthesis and growth but also integrates intracellular signaling into nutrient metabolism by promoting cell membrane translocation of GLUT4 and gene expression of glucokinase and FA synthase. In the absence of AKT, the expression levels of the genes responsible for glucose utilization and lipid synthesis were significantly reduced (47). AMPK, one of the downstream signals of AKT, acts as an energy sensor that regulates multiple physiological processes in the cardiovascular system and maybe a potential therapeutic target for HF. AMPK can similarly mediate the translocation of GLUT4, thereby increasing glucose uptake as a cardioprotective and adaptive response of the failed heart (48). AMPK also inhibits glycogen synthesis and promotes glucose oxidation (49). In addition, AMPK promotes the absorption and oxidation of FA. It increases the translocation of CD36 to the membrane and promotes FA uptake by cardiomyocytes. The Activated AMPK increases the CPT-1β activity by reducing malonyl-CoA production by inhibiting the ACC activity. Therefore, AMPK can increase FA oxidation (50). All of these effects ultimately increase ATP production to improve the imbalance between energy supply and energy demand in the failed heart. Moreover, previous studies reported that MCC950 exerts a protective effect by inducing autophagy through an AMPK-dependent mechanism (51). Consistent with previous reports, AKT activation was increased while AMPKα activation was inhibited after TAC surgery. The MCC950 treatment inhibited AKT activation and increased the phosphorylation levels of AMPKα. The effect of MCC950 on the AKT/AMPKα pathway may explain the changes in cardiometabolism, which needs further research.

This study has several limitations. We only explored the effect of MCC950 on metabolism after HF *in vivo*. Studies *in vitro* may help to explore the mechanism of the protective role of MCC950. In addition, the effects of MCC950 on HFD-induced metabolism dysfunction need further exploration.

## Conclusion

In conclusion, the most important finding is the protective role of MCC950 in alleviating HF induced by pressure overload in obese mice, and its mechanism is related to the improvement of cardiac metabolism. Our study provides a basis for the clinical application of NLRP3 inhibitors in obese patients with HF.

## Data Availability Statement

The original contributions presented in the study are included in the article/Supplementary Material, further inquiries can be directed to the corresponding author.

## Ethics Statement

The animal study was reviewed and approved by the Animal Care and Use Committee of Renmin Hospital of Wuhan University.

## Author Contributions

MW, MZ, and JYu contributed to the experimental design and wrote the manuscript. JL, JYe, ZW, and YX contributed to the acquisition and analysis of the data. ZZ, DY, YF, SX, WP, JZ, and JW reviewed the manuscript. All authors contributed to the article and approved the submitted version.

## Conflict of Interest

The authors declare that the research was conducted in the absence of any commercial or financial relationships that could be construed as a potential conflict of interest.

## Publisher’s Note

All claims expressed in this article are solely those of the authors and do not necessarily represent those of their affiliated organizations, or those of the publisher, the editors and the reviewers. Any product that may be evaluated in this article, or claim that may be made by its manufacturer, is not guaranteed or endorsed by the publisher.
