# Supplementary material for: MCC950, a Selective NLRP3 Inhibitor, Attenuates Adverse Cardiac Remodeling Following Heart Failure Through Improving the Cardiometabolic Dysfunction in Obese Mice
Source: Front Cardiovasc Med. 2022 May 12;9:727474. doi: 10.3389/fcvm.2022.727474 (PMC9133382; doi:10.3389/fcvm.2022.727474)

**Supplemental Table S1: Catalog numbers of antibodies.**

| **Antibody** | **Company** | **Catalog number** |
| --- | --- | --- |
| GAPDH | Abcam | AB8245 |
| α-SMA | Abcam | AB5694 |
| CD68 | Abcam | AB955 |
| CD206 | Abcam | AB64693 |
| CD36 | Abcam | AB133625 |
| GLUT4 | Abcam | AB188317 |
| COL1 | Proteintech | 66761-1-Ig |
| COL3 | Proteintech | 22734-1-AP |
| CPT1β | Proteintech | 22170-1-AP |
| t-ACC | Proteintech | 21923-1-AP |
| p-ACC | Proteintech | 29119-1-AP |
| MCD | Proteintech | 15265-1-AP |
| GLUT1 | Proteintech | 66290-1-Ig |
| t-PDH | Proteintech | 21176-1-AP |
| p-PDH | Proteintech | 29582-1-AP |
| PDK4 | Proteintech | 12949-1-AP |
| CASPASE-1 | Santa Cruz | SC-56036 |
| IL1β | Santa Cruz | SC-52012 |
| t-AKT | Cell Signaling Technology | 4685 |
| p-AKT | Cell Signaling Technology | 4060 |
| t-AMPKα | Cell Signaling Technology | 2532 |
| p-AMPKα | Cell Signaling Technology | 2535 |

**Supplemental Table S2:Primers for quantitative real-time PCR.**

| GENES | FORWARD (5' TO 3') | REVERSE (5' TO 3') |
| --- | --- | --- |
| GAPDH | AACTTTGGCATTGTGGAAGG | CACATTGGGGGTAGGAACAC |
| ANP | TACAGTGCGGTGTCCAACACAG | TGCTTCCTCAGTCTGCTCACTC |
| BNP | TCCTAGCCAGTCTCCAGAGCA | GGTCCTTCAAGAGCTGTCTCTG |
| β-MHC | CTGAAGGGCATGAGGAAGAGT | AGGCCTTCACCTTCAGCTGC |
| COL-1 | CCTCAGGGTATTGCTGGACAAC | CAGAAGGACCTTGTTTGCCAGG |
| COL-3 | TGCCACCCCGAACTCAAG | AGATCAGGCAGGGCCATAGCT |
| MMP-2 | GCCCCCATGAAGCCTTGTTT | TAGCGGTCTCGGGACAGAAT |
| MMP-9 | CCGACTTTTGTGGTCTTCCC | TTTGGAATCGACCCACGTCT |
| IL-1β | TGGACCTTCCAGGATGAGGACA | GTTCATCTCGGAGCCTGTAGTG |
| IL-18 | GACAGCCTGTGTTCGAGGATATG | TGTTCTTACAGGAGAGGGTAGAC |
| IL-10 | GCTCTTACTGACTGGCATGAG | CGCAGCTCTAGGAGCATGTG |
| PPARα | CACCATGGTGGACACAGAGAGCCCCATC | TTAGTACATGTCTCTGTAGATCTC |
| PGC1α | AATGCAGCGGTCTTAGCACT | TTTCTGTGGGTTTGGTGTGA |
| PGC1β | ATGTTCACAGCCCTTCGGTT | CACGGCATGCACTTCCATTT |
| CPT2 | TCCTCGATCAAGATGGGAAC | GATCCTTCATCGGGAAGTCA |
| MCAD | AGGGTTTAGTTTTGAGTTGACGG | CCCCGCTTTTGTCATATTCCG |
| SCAD | ACAGTGGATCACCCCTTTCAC | ACCCATGAGTCACCCTCTTCC |
| FATP1 | GGCCACCATTCCTACAGCAT | CCACCGTCAACCCGTAGATG |
| FABP4 | AAGAAGTGGGAGTGGGCTTT | TCGACTTTCCATCCCACTTC |
| FABP5 | CAAAACCGAGAGCACAGTGA | TTTGACCGCTCACTGAATTG |
| CD36 | TCTCCCTGAAGCCAATCT | GTTTTCTCGCCAACTCCC |
| GLUT1 | GGGAGAGGTGTCACCTACAGC | ATTGCCCATGATGGAGTCTAA |
| GLUT4 | GATTCTGCTGCCCTTCTGTC | ATTGGACGCTCTCTCTCCAA |
| PDK4 | AAAGATGCTCTGCGACCAGT | CCTGCTTGGGATACACCAGT |
| UCP2 | TCGAAGCCTACAAGACCATTGCAC | ACCAGCTCAGCACAGTTGACAATG |

**Supplemental Table S3: Echocardiographic parameters in mice of each group.**

| **Groups** | **Sham+vehicle** | **Sham+MCC950** | **TAC+vehicle** | **TAC+MCC950** |
| --- | --- | --- | --- | --- |
| HR(bpm) | 455±23 | 453±19 | 451±29 | 451±30 |
| LVEDs (mm) | 2.5±0.2 | 2.7±0.1 | 3.6±0.4^*^ | 2.9±0.2^#^ |
| LVEDd (mm) | 3.8±0.3 | 4.1±0.1 | 4.7±0.4^*^ | 4.1±0.2^#^ |
| IVSs (mm) | 1.08±0.10 | 1.06±0.10 | 1.41±0.20^*^ | 1.1±0.10^#^ |
| IVSd (mm) | 0.63±0.10 | 0.68±0.10 | 0.90±0.10^*^ | 0.75±0.03^#^ |
| LVPWs (mm) | 1.06±0.19 | 1.14±0.10 | 1.46±0.12^*^ | 1.2±0.15^#^ |
| LVPWd (mm) | 0.61±0.08 | 0.68±0.09 | 0.83±0.12^*^ | 0.76±0.16^#^ |
| EF (%) | 70.2±3.5 | 69.4±4.0 | 50±3.5^*^ | 59.8±4.2^#^ |
| FS (%) | 34.4±2.4 | 33.8±2.9 | 21.7±1.7^*^ | 27.3±2.7^#^ |

LVEDs, LV end-systolic diameter; LVEDd, LV end-diastolic diameter; IVSs, interventricular septal thickness at end-systole; IVSd, interventricular septal thickness at end-diastole; LVPWs, LV posterior wall thickness at end-systole; LVPWd, LV posterior wall thickness at end-diastole; EF, ejection fraction; FS, fractional shortening. ^⁎^P < 0.05 vs sham+vehicle group; ^#^P < 0.05 vs TAC+vehicle group.

**Supplementary Figure s1:** **study profile**


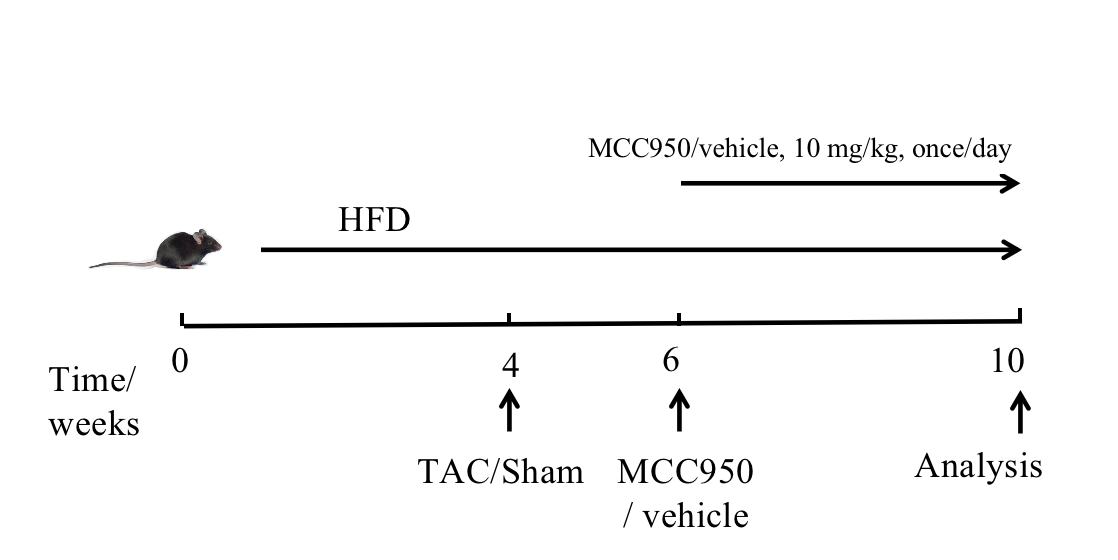

Supplement: Supplementary file 1 [file Data_Sheet_1.DOCX]
